# Perindopril: do randomised, controlled trials support an ACE inhibitor class effect? A meta-analysis of clinical trials

**Published:** 2009-04

**Authors:** Jacques R Snyman, Francois Wessels

**Affiliations:** Department of Pharmacology, Faculty of Health Sciences, University of Pretoria, Pretoria; Outcomes, Consultants, Pretoria

## Abstract

**Background:**

Due to the lack of face-to-face trials between ACE inhibitors, clinicians and third-party funders may assume they provide similar outcomes. As a result, ACE inhibitors may be prescribed interchangeably and deemed to provide the same outcomes for all patients when used chronically, that is for more than six months.

**Objective:**

This meta-analysis aims to dispute the assumption of a class effect when prescribing ACE inhibitors (ACEIs), since the evidence from all the clinical trials is not uniform and therefore a direct comparison is impossible.

**Methods:**

Published randomised, controlled trials were selected using an applicable literature search for all ACEIs, irrespective of drug combination, for any cardiovascular outcome (both composite and individual outcomes were included). The average length of ACEI exposure per trial had to be longer than six months). This meta-analysis was performed using odds ratios as the parameter of efficacy in a fixed-effects model.

**Results/Conclusion:**

Perindopril resulted in significantly fewer patients reaching the primary endpoint versus all other ACEIs combined. The results were consistent for myocardial infarction, stroke and mortality (5 vs 11%, *p* = 0.0001). Perindopril alone or as part of combination therapy in clinical trials seemed to deliver clear and consistent outcome differences compared to other ACEI trials. In the presence of positive outcomes from robust randomised, controlled trials for perindopril, one cannot assume a class effect for all ACEIs.

## Summary

Angiotensin-converting enzyme (ACE) inhibitors are a treatment option selected for patients throughout the cardiovascular continuum. 1,2 In patients with hypertension, guidelines throughout the world recommend an ACEI as one of the first-line therapies. Compelling indications for the use of ACEIs in these guidelines are listed in Table 1.^3^

**Table 1:** Recommendations On Compelling Indications For A Specific Drug Class^3^

Any drug that lowers BP unless absolutely contraindicated, will conffer protection against target-organ damage. However, the following classes of drugs have additional protective properties in the case of the listed associated clinical conditions/target-organ damage.	
*Compelling indications*	*Drug class*
Angina	Beta-blocker or CCB (rate lowering preferred)
Prior myocardial infarct	Beta-blocker and ACEI (ARB if ACEI intolerant). Verapamil if beta-blockers contraindicated and aldosterone antagonist. Loop diuretics for volume overload
Left ventricular hypertrophy (confirmed by ECG)	ARB (preferred) or ACEI
Stroke: secondary prevention	Low dose thiazide-like diuretic and ARB or ACEI
Diabetes type 1 or 2 with or without evidence of microalbuminuria or proteinuria	ACEI or ARB – usually in combination with a diuretic
Chronic kidney disease	ACEI or ARB – usually in combination with a diuretic
Isolated systolic hypertension	Low-dose thiazide or thiazide-like diuretic or long-acting CCB

These guidelines therefore leave the impression that the clinical benefit across the spectrum of ACEI molecules may be taken as uniform. This leads the clinician to believe that the outcomes from an array of diverse randomised, controlled trials are the same, despite significant differences in design criteria (inclusion and exclusion criteria, dosage and dosing intervals, etc).

The aim of this meta-analysis was to compare the magnitude of clinical benefit across the spectrum of ACEI molecules, irrespective of trial design and dosage, since this is usually the way in which these products are used in clinical practice. It was therefore noted that ACEIs are prescribed in combination with other drugs to treat cardiovascular conditions in approximately 80% of patients,^4^ making it illogical to try and separate the effect of combinations from that of single molecules, since the clinical benefit is the ultimate driver of how the patient should be treated.

It is well established that a broad range of blood pressure-lowering drugs, including ACEIs, reduce the risks of major cardiovascular (CV) events. Various meta-regression analyses and overviews of trials have determined the benefits of blood pressure lowering.^5^-^7^ The conclusions made in ACEI trials (i.e. ACEI vs angiotensin receptor blockers, calcium channel blockers, diuretics and β-blockers) have further emphasised the need for ACEIs to be part of standard therapy, not only for hypertension but for the majority of cardiovascular conditions.^5^,^6^,^8^,^9^ However, no formal attempts have been made to evaluate the contribution of one or more ACEIs to the specific value of the outcomes achieved by the class.

This analysis focuses on the contribution of perindopril to the overall benefits seen in the ACEI class, due to the large number of positive clinical trials utilising perindopril. It has already been established that there is sufficient clinical data demonstrating the proven clinical benefit of perindopril in various cardiovascular conditions. This meta-analysis was undertaken to determine whether perindopril produces a greater reduction in cardiovascular events and/or morbidity/mortality outcomes for all patients versus all the other commonly prescribed ACEIs combined as a class. The main aim of this analysis was therefore to compare the ACEI trials that delivered tangible clinical outcomes (irrespective of type of outcome).

Applying the results of clinical trials to general practice is extremely difficult. This analysis, by pooling the clinical trial data, will allow clinicians to discern which molecule has the most robust clinical data to support its use in any patient within the cardiovascular continuum.

## Methods

The following trials were considered for inclusion: all randomised, controlled trials of ACEI therapy (placebo and/or other active therapy) for any cardiovascular outcomes. A literature search was conducted using Pubmed, Medline and Cochrane library. Relevant articles were selected on the basis of titles, references cited in reviews and commentaries, and selected publications. The rationale for selection of these trials was to determine whether the ACEI arm of the trial had an effect on the clinically stated outcomes irrespective of comparator, blood pressure lowering or patient-specific characteristics. The fact that baseline criteria differed among the studies is acknowledged, however this was a drawback of all indirect comparisons. In the absence of uniformity, this analysis focuses on clinical outcomes as the driver of the comparison, since this ultimately informs clinical practice and is the only guarantee of benefit. It is therefore acknowledged that this analysis is not typical, in that the baseline demographics differ, but this is necessarily due to the lack of suitable comparable studies.

All studies of a minimum of six months’ duration that measured a specific ACEI outcome effect, both composite and individual cardiovascular measures, were included in the analysis. Studies measuring only surrogate endpoints were not considered.

**Fig. 1.**
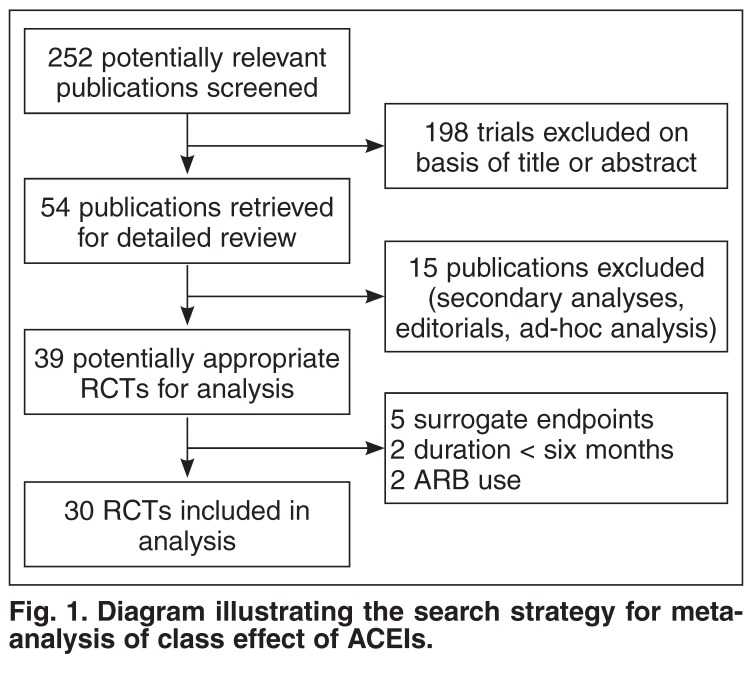
Diagram illustrating the search strategy for metaanalysis of class effect of ACEIs.

The qualifying studies were checked for: blinding, randomisation, completeness of follow up, and methods of measuring outcome events. All trials had to have reported at least one of the pre-specified outcomes (all-cause mortality, stroke, CV events, mortality due to CV events, and myocardial infarction) and had to have had at least six months’ follow up. Due to large discrepancies in the primary outcomes, an analysis was performed using the primary outcome of the trials, irrespective of these differences, i.e. was the stated outcome reached or not? (see Fig. 1 for search strategy).

The meta-analysis was performed using odds ratios (ORs) as the parameter of efficacy, utilising a fixed-effects model. This is a measure of treatment effectiveness and also examines the effects of other variables in that relationship using logistic regression. The influence of the perindopril studies was assessed on the pooled effect sizes by excluding these studies in a manner similar to the Tobias method.^10^ This measured the influence of the perinodopril studies against the combined ACEI effect using Statsdirect software.

## Results

The initial literature search resulted in 253 studies, but outcome data were available for only 30 trials that met the inclusion criteria. These trials in total included approximately 204 000 patients (see Table 2 for demographic data).

**Table 2:** Trials Included In Meta-Analysis

*Acronym*	*Name*	*Comparison (duration of follow up)*	*Primary endpoint RR (p value)*	*Patients*
ASCOT^11^	Anglo-Scandinavian Cardiac Outcomes Trial	Atenolol ± thiazide (bendroflumethiazide vs amlodipine ± perindopril (5.5 years)	Composite: a,b 10%; *p* = 0.1052*	19 257
EUROPA^12^	The EURopean trial On reduction of cardiac events with Perindopril in stable coronary Artery disease	Perindopril 8 mg vs placebo (4.2 years)	Composite: a,b,f 20%; *p* = 0.0003	12 218
PROGRESS^13^	Perindopril protection aGainst REcurrent Stroke Study	Perindopril 4 mg ± indapamide vs placebo (3.9 years)	Fatal/non-fatal stroke 28%; *p* < 0.0001	6 105
PEP-CHF^14^	Perindopril in Elderly People with Chronic Heart Failure	Perindopril 4 mg vs placebo in elderly (1 year)	Composite: a,d 31%; *p* = 0.055	850
STOP-2^15^	Swedish Trial in Old Patients with hypertension-2 study	Atenolol, metoprolol, pindolol or HCTZ + amiloride vs enalapril, lisinopril, isradipine (4.5 years)	Composite: a,b,c,f 1%; *p* = 0.89, NS	6 614
ANBP2^16^	2nd Australian National Blood Pressure study group	ACE inhibitors vs diuretics (enalapril vs HCTZ) (5 years)	Composite: a,b,f 11%; *p* = 0.05, NS	6 083
JMIC-B^17^	Japan Multicentre Investigation for Cardiovascular diseases – B	Enalapril 5–10 mg/day or lisinopril 10–20 mg/day vs Nifedipine Retard 10–20 mg bd	Composite: a,b,d,f 1.05; *p* = 0.75, NS	1 650
SCAT^18^	Simvastatin/enalapril Coronary Atherosclerosis Trial	Simvastatin vs enalapril vs placebo (47.8 months)	Composite: a,b,c,d,e NS	460
ALLHAT^9^	Antihypertensive and Lipid-Lowering treatment to prevent Heart Attach Trial	Chlorthalidone vs amlodipine vs lisinopril (6 years)	Composite: b,f NS, *p* = 0.71	33 357
HOPE^19^	Heart Outcomes and Prevention Evaluation study	Ramipril 10 mg vs placebo (4.5 years)	Composite: a,b,c 25%; *p* < 0.001	9 297
PART 2^20^	Prevention of Atherosclerosis with Ramipril Trial	Ramipril 5–10 mg vs placebo (4.7 years)	Composite: a,b,c NS	617
QUIET^21^	QUinapril Ischemic Event Trial	Quinapril 20 mg vs placebo (3 years)	Composite: a,b,d,e,f RR 1.04; *p* = 0.6, NS	1 750
ABCD^22^	Appropriate Blood pressure Control in Diabetes	Nisoldipine vs enalapril (5 years)	Fatal/non-fatal MI risk ratio 9.5; no *p* value, NS	470
CAMELOT^23^	Comparison of AMlodipine vs Enalapril to Limit Occurences of Thrombosis	Amlodipine or enalapril vs placebo (24 months)	Composite: a,b,c,d,e,f 15%; *p* = 0.16, NS	1 991
AIRE^24^	Acute Infarction Ramipril Efficacy Study	Ramipril 5 mg vs placebo (15 months)	All-cause mortality 27%; *p* = 0.002	2 006
INVEST^25^	INternational VErapamil SR/ trandolopril STudy	Verapamil vs atenolol (plus HCTZ and/or trandolopril) (24 months)	Composite: a,b,c 2%; *p* = 0.57, NS	22 576
TRACE^26^	TRAndolapril Cardiac Evaluation study	Trandolapril vs placebo (24–50 months)	Death – all cause 22%; *p* = 0.001	1 749
PEACE^27^	Prevention of Events with Angiotensin Converting Enzyme inhibition trial	Trandolapril vs placebo (4.8 years)	Composite: a,b,e 4%; *p* = 0.43, NS	8 290
PREAMI^28^	Perindopril and Remodelling in Elderly with Acute Myocardial Infarction study	Perindopril 8 mg vs placebo (12 months)	Composite: a,d,f 38%; *p* < 0.001	1 252
CONSENSUS I^29^	Co-Operative North Scandinavian ENalapril SUrvival Study	Enalapril vs placebo in severe heart failure (up to 20 months)	Mortality 31%; *p* = 0.001	253
CONSENSUS II^30^	Co-Operative North Scandinavian ENalapril SUrvival Study II	Enalapril vs placebo (6 months) (stopped early)	Mortality NS	6 090
SOLVD I^31^	Studies Of Left Ventricular Dysfunction	Enalapril vs placebo (> 3 years)	Mortality 16%; *p* = 0.0036	4 228
V-HeFT^32^	Vasodilator Heart Failure Trial	Hydralazine/isosorbide dinitrate vs enalapril 20 mg (2.3 years)	Mortality 28%; *p* = 0.016	804
GISSI 3^33^	Gruppo Italiano per lo Studio della Sopravvivenza nell’Infarto Miocardico	Lisinopril vs transdermal glycerol trinitrate (GTN) vs combination (6 months)	Mortality 6%; *p* = 0.03	18 895
DREAM^34^	Diabetes Reduction Assessment with ramipril and rosiglitazone Medication trial	Ramipril vs ramipril + rosiglitazone vs placebo (3 years)	Composite: a,f 9%; *p* = 0.15, NS	5 269
CAPPP^35^	CAPtopril Prevention Project	Captopril vs atenolol/ bendroflumethiazide (6.1 years)	Composite: a,b,c RR 1.05; *p* = 0.52, NS	10 985
DIABHYCAR^36^	type 2 DIABetes, Hypertension, CArdiovascular events and Ramipril study	Ramipril vs placebo in type 2 diabetes (4 years)	Composite: a,b,c,f HR 1.03; *p* = 0.65, NS	4 912
UKPDS^37^	UK Prospective Diabetes Study group	Captopril vs atenolol (11.1 years)	Mortality NS	758
SAVE^38^	Survival and Ventricular Enlargement Trial	Captopril vs placebo (42 months)	Mortality 19%; *p* = 0.019	2 231
ADVANCE^6^	Action in Diabetes and Vascular Disease: Preterax and Diamicron MR Controlled Evaluation Trial	Perindopril + indapamide vs placebo (4.3 years)	Composite: a,b,c,f 9%; *p* = 0.04	11 140

Composite endpoints: a = mortality, b = fatal/non-fatal MI, c = stroke, d = hospitalisation, e = revascularisation, f = other. NS = non-significant difference in outcome. *ASCOT study stopped early due to 11% risk reduction in all-cause mortality, *p* = 0.0247 – safety board halted study.

Seven ACEIs were researched in the included studies, irrespective of dosages and patient characteristics. The primary endpoint of all trials differed in some respects, but the majority were composite endpoints. Composite endpoints lead to higher event rates and enable smaller sample sizes and shorter followup, or both. These endpoints may prove challenging to interpret but it is precisely this result that should be considered in making an evidence-based selection. All the primary endpoints were combined in the initial analysis. Wherever possible, especially in three-arm treatment design trials, both comparators were included in order to provide each ACEI with the maximum possible benefit of effect.

## Comparison of primary endpoints

In these event-driven trials, the ACEIs as a class had a lower likelihood of an event occurring, compared to any of the comparator drugs or placebo (OR 0.91; 95% CI: 0.88–0.94; *p* < 0.0001). This primary endpoint analysis was only for composite endpoints. (Trials with specific primary endpoints, e.g. stroke, myocardial infarction, are analysed in that specific section.) Only four of the trials produced a statistically significant reduction in the primary clinical endpoint: three trials using perindopril and one using ramipril.

**Fig. 2.**
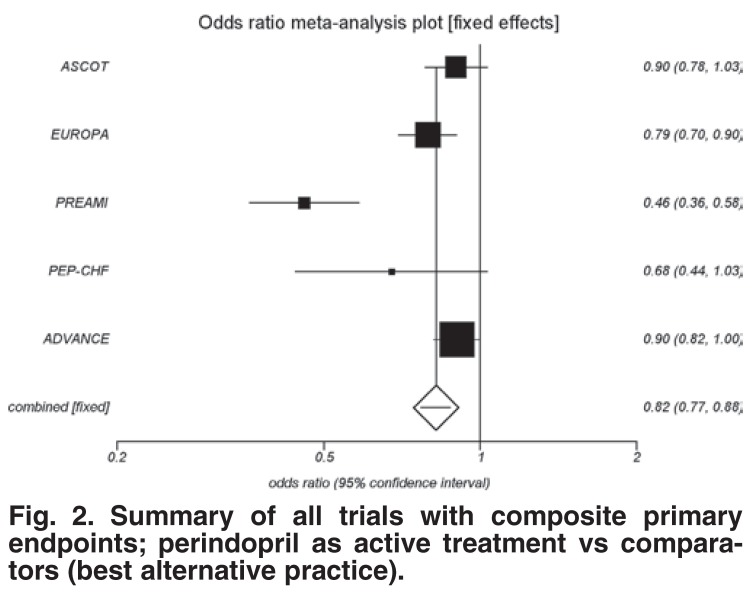
Summary of all trials with composite primary endpoints; perindopril as active treatment vs comparators (best alternative practice).

The effect size for perindopril alone was larger than that of the combined ACEI analysis. See Fig. 2 for details. Perindopril showed a significant risk reduction of 18% (OR 0.82; 95% CI: 0.77–0.88; *p* < 0.0001) when compared to the overall ACEI effect.

**Fig. 3.**
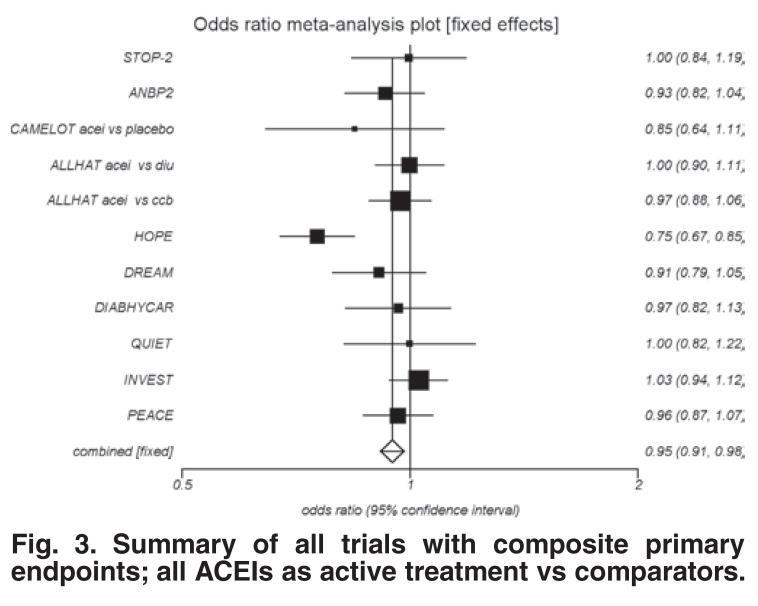
Summary of all trials with composite primary endpoints; all ACEIs as active treatment vs comparators.

When this analysis was repeated, excluding the perindopril studies, the ACEI effect was reduced to 5% (OR 0.95; 95% CI: 0.91–0.98; *p* = 0.0039) (Fig. 3). It is clear that the perindopril outcomes drove the magnitude of the ACEI benefit.

## Myocardial infarction as outcome

In all the trials, only 18 reported an actual measure of myocardial infarction (MI) as a specific outcome. No separate analysis of fatal and non-fatal MI was performed, as most studies opted to group the two outcomes, or selected and reported on only one. A subgroup of each incident would therefore reduce the statistical power and overall impact, defying the aim of this analysis.

**Fig. 4.**
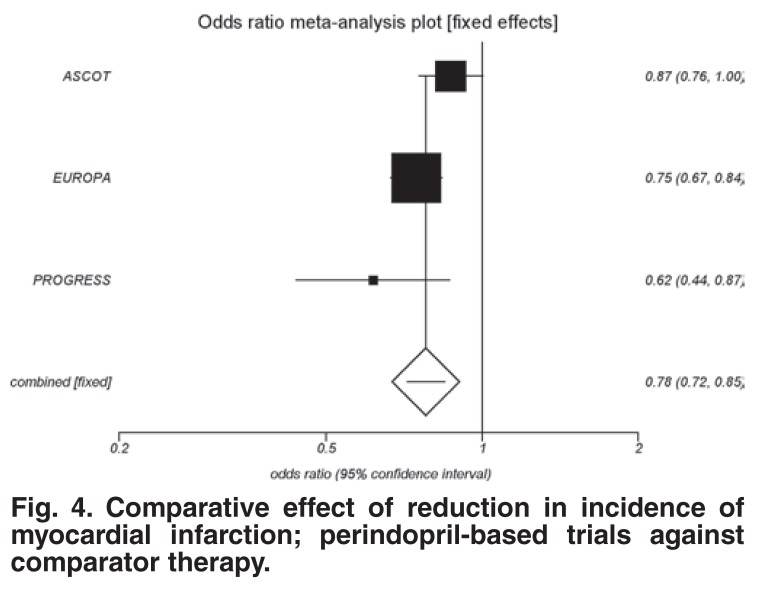
Comparative effect of reduction in incidence of myocardial infarction; perindopril-based trials against comparator therapy.

The perindopril trials demonstrated a highly significant event reduction (OR 0.78; 95% CI: 0.72–0.85; *p* < 0.0001 (Fig. 4). This was better than the event reduction with all other ACEIs combined (OR 0.86; 95% CI: 0.80–0.91; *p* < 0.0001). Perindopril resulted in nearly twice as many events saved. The difference in absolute risk in effect was 0.32 for ACEIs and 1.45 for perindopril (*p* < 0.0001).

The only other ACEI that has demonstrated a benefit in reduction of MI is ramipril, as used in the HOPE trial.^19^ This reduction was similar in magnitude to the perindopril effect but has only been proven in high-risk CAD patients.

## Stroke as outcome

**Fig. 5.**
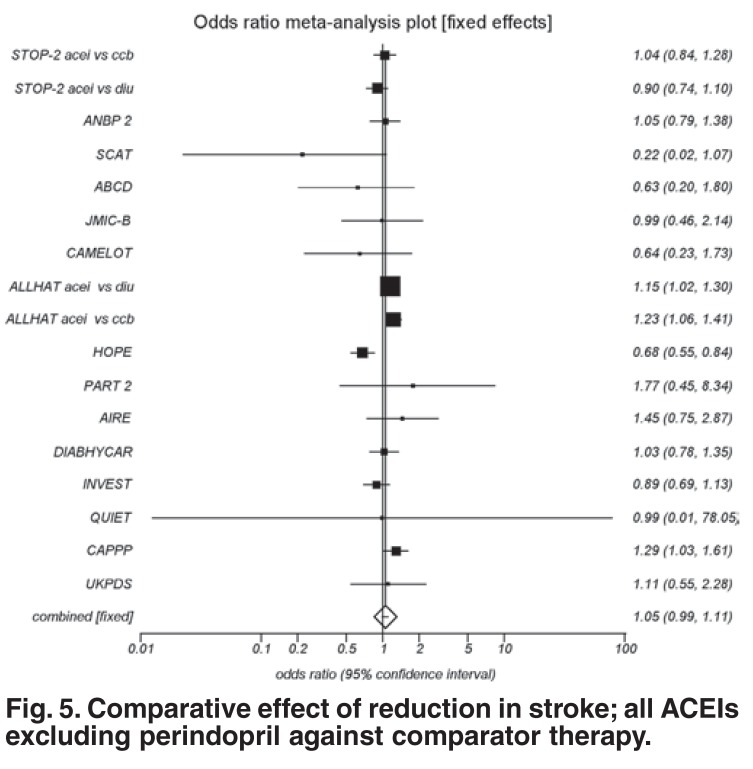
Comparative effect of reduction in stroke; all ACEIs excluding perindopril against comparator therapy.

This current analysis confirms previous results,^5^,^39^ in that when the ACEIs were pooled, the overall effect resulted in a risk reduction (OR 0.96; *p* = 0.0451). The effect of perindopril on stroke demonstrated a highly significant reduction in event rate (OR 0.79; 95% CI: 0.72–0.86; *p* < 0.0001). However, the exclusion of perindopril trials from the other ACEI trials reproduced a nonsignificant reduction in stroke events (OR 1.05; *p* = 0.1287) (Fig. 5 compared to comparator drugs).

In pooling all the evidence for stroke reductions with ACEIs, the effect of perindopril became diluted. Therefore, from the data set analysed, all ACEIs cannot be advocated to reduce the risk of stroke. This reconfirms findings that lowering blood pressure is important but that the particular drug properties and dosages may be even more relevant.

## Mortality as outcome

Mortality is the most commonly measured outcome in cardiovascular trials (and probably the most critical), although in many instances the causes for death are not analysed homogeneously. In order to produce an objective, meaningful analysis, all causes of mortality were combined; there was no separation of CV death, death due to stroke or MI, or all-cause mortality. It is well accepted that additional risk factors and co-morbid diseases may compound the measurement of this outcome, but for the patient, it remains immaterial and yet critically important. This should also be true for the treating clinician.

**Fig. 6.**
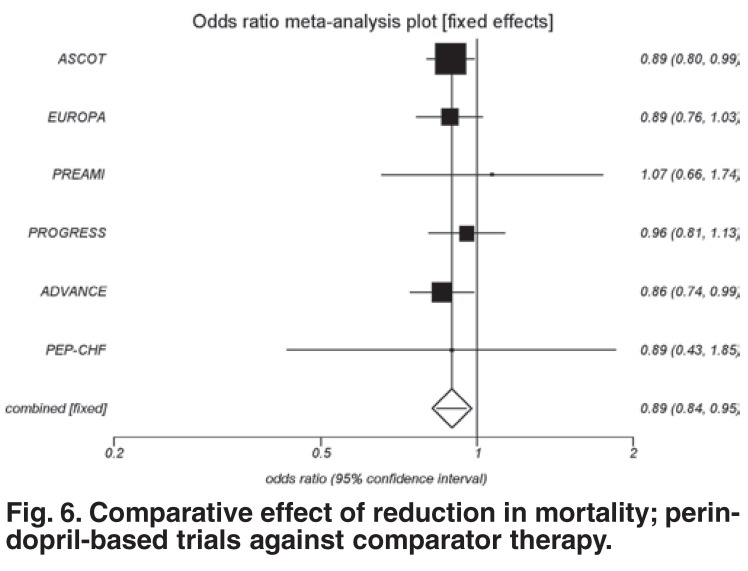
Comparative effect of reduction in mortality; perindopril-based trials against comparator therapy.

Perindopril, in the six outcome trials, showed a reduction in death compared to other drugs and or placebo (OR = 0.89; 95% CI: 0.84–0.95; *p* = 0.0008) (Fig. 6). This represents a significant 11% reduction in mortality, which is both a clinically and statistically significant benefit for all cardiovascular patients. This outcome includes all patients, irrespective of entry criteria (i.e. diabetes, cerebrovascular incident, hypertension, post-myocardial infarction or high risk for cardiovascular disease based on a combination of risk factors).

The ACEI benefit, demonstrated in 23 different ACEI trials, excluding perindopril, was only 5% (OR 0.95; 95% CI: 0.92–0.98; *p* = 0.0033). The absolute risk difference correlates to a NNT of 210 for ACEIs versus 127 for perindopril (*p* < 0.0001).

## Discussion

Only two other analyses have been performed to compare ACEI effects as a class of drugs.^40^,^41^ Both these articles by Pilote used ramipril as the comparator and Canadian retrospective data. The conclusions drawn indicated that survival benefits ‘differed according to specific ACE inhibitor prescribed’.

In this meta-analysis of patients (*n* = 204 000) treated with ACEIs for different conditions in the cardiovascular continuum, the results clearly demonstrated a significant reduction in events: all-cause events (primary endpoints), mortality, stroke and myocardial infarction. According to this analysis of published clinical data, it would seem that the effects of perindopril were, however, larger than those of the rest of the class of ACEIs. The magnitude of this effect should be quantified in subsequent analyses.

It is accepted that there are differences in outcomes that may be related to the differences in baseline risk and the use of concomitant secondary medications such as statins, β-blockers and aspirin. Consideration is given to differences in design, for example, the ALLHAT study where no diuretic could be added to the ACEI. It is statistically impossible to discern individual drug effects within a trial; this does not imply a lack of effect nor does it indicate probable effect. These factors were not compensated for in this analysis due to the complexity of the multiple trial designs and the lack of comparable data. This does not detract from the outcome since the ACEI benefit was obvious in spite of the other drugs being used in all arms of the trials included, i.e. best-alternative care, and only strengthens the benefit observed with the ACEI. Clinicians are left with a clinical decision to select a drug from a class and should select based on proven outcome benefit rather than ‘presumed’ benefit.

In the face of conclusive evidence, science dictates that positive outcomes should be considered. The inference of outcomes between two drugs within the same class is not ideal; however in the absence of head-to-head trials, a level of informal comparison must be made. The obvious ideal solution to the dilemma of ‘class effect’ is a head-to-head study with comparable doses driving similar surrogate targets (e.g. blood pressure lowering) with sufficient patient numbers, but the realisation is that this will never occur within the ACEI class, mainly due to the cost implications involved.

It is more critical to view the results of multiple trials with relevant agents as being in some way attributable to the individual properties of the ACEIs, despite there being no obvious proven relationship between the pharmacological properties (e.g. half life, tissue affinity and lipophilicity) of the individual medications and their clinical benefit in the trials. The difference in the magnitude of positive outcomes achieved with specific agents compels us to appreciate these pharmacological differences and argues against a class effect.

Several recent publications have attempted to analyse the ACEI properties. The latest by Comini concluded: ‘Our findings provide further proof of differential effects associated with ACE inhibitor therapy and suggest that the clinical benefits associated with these drugs may not solely reflect a class effect extending their benefit beyond blood pressure lowering effect’.^42^

The findings from the current analyses are complemented by various other published analyses such as Pilote,^40^ who found: ‘In summary, our results suggest that not all drugs within the class of ACE inhibitors should be considered to have the same effect. The exact mechanisms causing these differences are unclear, although they are probably related to the structural and pharmacological characteristics of the individual drugs’.

Meta-analyses provide a framework to formally evaluate the treatment effect quantitatively from at least two trials. However, the Committee for Proprietary Medicinal Products guidance document on meta-analyses states that a meta-analysis involving trials that are not convincing in their own right is inferior to one robust trial supported by smaller trials. From a statistical point of view, it is important to evaluate the possibility of a qualitative trial-by-treatment interaction, and to be aware that differential exposure to study medication across different trials can have an effect on outcome.^43^ This clearly confirms that all the perindopril trials with robust clinical outcomes should be considered to be more valuable than the other ACEI trials with less-than-convincing outcomes.

In order to make any meaningful comparison of drug usage, the doses of the ACEIs need to be considered. It is evident that in many trials, the positive outcomes were only achieved when high dosages of ACEIs were used. This was confirmed by the high-dose ramipril in high-risk patients in the HOPE study. Also, when using enalapril or lisinopril, the only doses with confirmed outcomes were in excess of 40 mg (SOLVD I,^32^ CONSENSUS I^30^ studies).

Perindopril’s outcomes were achieved over the entire dose spectrum, depending on the specific outcome tested (4–8 mg).^7^,^11^-^14^,^28^ However, when outcomes were positive in the high-risk patients, the dosage of perindopril used was also at the top end of the dose range (EUROPA^12^) (8 mg). This is re-emphasised in the ADVANCE study,^7^ where 50% of patients were on 4 mg perindopril and 50% on 8 mg perindopril. ADVANCE measured the effect of high-dose perindopril in reducing outcomes.

## Comparable outcomes with most commonly used ACEIs: enalapril and lisinopril

The results of the meta-analysis isolating the enalapril and lisinopril results are summarised in Table 3. Comparing perindopril with enalapril/lisinopril, the magnitude of benefit is obvious: MI reduction was the only significant outcome for enalapril/lisinopril, which translates into an ARR of 0.40* versus absolute risk reduction (ARR) or 1.45 for perindopril. This means that to reduce one event, 250 patients need treatment with enalapril/lisinopril versus only 69 with perindopril. (*Calculated from data obtained from STOP2, ALLHAT, ANBP2, ABCD and CAMELOT studies.)

**Table 3:** Enalapril/Lisinopril Studies Compared To Perindopril Outcomes: OR

*Outcome*	Enalapril/lisinopril*	*Perindopril*
Primary endpoint (any event)	0.97; *p* = 0.2085	0.82; *p* < 0.0001
Myocardial infarction	0.82; *p* = 0.0001	0.78; *p* < 0.0001
Stroke	1.05; *p* = 0.412	0.79; *p* < 0.0001
Mortality	0.99; *p* = 0.8466	0.89; *p* = 0.0008

*This analysis combined the results of both drugs, as in at least two trials, either drug could be used or no separation of results can be performed. From these trials it is evident that their use would be synonymous or deemed equivalent.

The other outcomes for enalapril/lisinopril were not significant, whereas perindopril maintained the same order of benefit (NNT: 60, 63, 124, respectively for composite outcome, stroke, and mortality**). Since the cost of a single event is generally high, this clearly offsets any price difference between drugs. (**Calculated from data obtained from ASCOT, EUROPA and PROGRESS studies.) The absolute differences between perindopril and enalapril/lisinopril translate into a two- to three-fold reduction in outcomes.

Similarly, ramipril effects should be considered in their entirety with DREAM, DIABHYCAR, PART-2, AIRE and HOPE studies all being combined. This effect would be driven entirely by the outcomes of HOPE – high dose (10 mg) at night in high Similarly, ramipril effects should be considered in their entirety with DREAM, DIABHYCAR, PART-2, AIRE and HOPE studies all being combined. This effect would be driven entirely by the outcomes of HOPE – high dose (10 mg) at night in high-risk CAD patients. Application of these results would have been appropriate in this set of patients only, and would have been extrapolated to hypertensive or other cardiovascular patients.

In a recent publication by Hansen,^44^ the ACEIs were shown to demonstrate a similar clinical efficacy after myocardial infarction. The conclusion that there is a class effect is based on the treatment post MI in the acute setting and when used in comparable dosages. In the clinical data from the EUROPA^12^ study, where 65% of patients had a history of MI, the use of 8 mg perindopril in this chronic setting demonstrated a reduction in the primary endpoint of major cardiac events, especially MI. The conclusion that ‘the dosage used appears to influence clinical efficacy, and using appropriate dosage is thus important to achieve full benefits of treatment’ is of paramount importance. Assuming a class effect of ‘comparable dosages’ is therefore flawed, as no dosage comparisons exist across the class of ACEIs. Therefore, only the proven clinically effective dosages of specific ACEIs should be used.

## Conclusion

This overview of cardiovascular studies has clearly confirmed the hypothesis that trials utilising perindopril have consistently and convincingly demonstrated the clinical benefit of using this ACEI. The clinical outcomes from individual trials, as well as in a meta-analysis format, have proven the lack of a so-called class effect within the ACEI class. The point estimates of all the combined perindopril trials lie outside the CI of the overall estimate of all the ACEIs, indicating the excessive influence. It is evident that perindopril and, to a lesser extent, ramipril have good clinical outcomes, warranting their selection over any other ACEI.

Caution has to be used in the interpretation of the results, as many of the outcome studies, which met the search and methodological criteria of the meta-analysis, involved use of perindopril in varying dosages and in combination with a variety of other drugs. It is impossible to attribute all the benefits achieved solely to the effect of the ACEI. Used in combination with amlodipine or indapamide in different clinical settings demonstrated the proven benefit. This meta-analysis merely confirmed the effect that multiple therapies can achieve in various cardiovascular clinical settings. The use of perindopril alone or in combination with amlodipine and indapamide all contributed to the positive effects shown. The positive effects of indapamide and amlodipine individually may also have contributed to the positive results and would need to have a separate analysis to disprove.

Based on this meta-analysis, the assumption that a class effect exists for all ACEIs may not be the most correct option. To therefore knowingly recommend an ACEI that has no conclusive clinical outcomes data in any of the cardiovascular conditions discussed may need to be reviewed and reconsidered.
